# Interaction between the genetic risk score and dietary protein intake on cardiometabolic traits in Southeast Asian

**DOI:** 10.1186/s12263-020-00678-w

**Published:** 2020-10-12

**Authors:** Sooad Alsulami, A. S. Aji, U. Ariyasra, S. R. Sari, N. Tasrif, F. F. Yani, J. A. Lovegrove, I. R. Sudji, N. I. Lipoeto, K. S. Vimaleswaran

**Affiliations:** 1 Department of Clinical Nutrition, Faculty of Applied Medical Sciences, King Abdulaziz University, Jeddah, Saudi Arabia; 2 Hugh Sinclair Unit of Human Nutrition, Department of Food and Nutritional Sciences, University of Reading, PO Box 226, Whiteknights, Reading, RG6 6AP UK; 3 Postgraduate Department of Biomedical Science, Faculty of Medicine, Andalas University, Padang, West Sumatra Indonesia; 4 Department of Nutrition, Faculty of Health Science, Universitas Alma Ata, Yogyakarta, Indonesia; 5 Public Health Department, Faculty of Medicine, Andalas University, Padang, Indonesia; 6 Department of Child Health, Faculty of Medicine, Andalas University, Padang, Indonesia; 7 Institute for Cardiovascular and Metabolic Research (ICMR), University of Reading, Reading, UK; 8 Biomedical Laboratory, Faculty of Medicine, Andalas University, Padang, Indonesia; 9 Department of Nutrition, Faculty of Medicine, Andalas University, Padang, Indonesia

**Keywords:** GRS, BMI, WC, Triglyceride, Interaction, Indonesian

## Abstract

**Background:**

Cardiometabolic diseases are complex traits which are influenced by several single nucleotide polymorphisms (SNPs). Thus, analysing the combined effects of multiple gene variants might provide a better understanding of disease risk than using a single gene variant approach. Furthermore, studies have found that the effect of SNPs on cardiometabolic traits can be influenced by lifestyle factors, highlighting the importance of analysing gene-lifestyle interactions.

**Aims:**

In the present study, we investigated the association of 15 gene variants with cardiometabolic traits and examined whether these associations were modified by lifestyle factors such as dietary intake and physical activity.

**Methods:**

The study included 110 Minangkabau women [aged 25–60 years and body mass index (BMI) 25.13 ± 4.2 kg/m^2^] from Padang, Indonesia. All participants underwent a physical examination followed by anthropometric, biochemical and dietary assessments and genetic tests. A genetic risk score (GRS) was developed based on 15 cardiometabolic disease-related SNPs. The effect of GRS on cardiometabolic traits was analysed using general linear models. GRS-lifestyle interactions on continuous outcomes were tested by including the interaction term (e.g. lifestyle factor*GRS) in the regression model. Models were adjusted for age, BMI and location (rural or urban), wherever appropriate.

**Results:**

There was a significant association between GRS and BMI, where individuals carrying 6 or more risk alleles had higher BMI compared to those carrying 5 or less risk alleles (*P* = 0.018). Furthermore, there were significant interactions of GRS with protein intake on waist circumference (WC) and triglyceride concentrations (*P*_interaction_ = 0.002 and 0.003, respectively). Among women who had a lower protein intake (13.51 ± 1.18% of the total daily energy intake), carriers of six or more risk alleles had significantly lower WC and triglyceride concentrations compared with carriers of five or less risk alleles (*P* = 0.0118 and 0.002, respectively).

**Conclusions:**

Our study confirmed the association of GRS with higher BMI and further showed a significant effect of the GRS on WC and triglyceride levels through the influence of a low-protein diet. These findings suggest that following a lower protein diet, particularly in genetically predisposed individuals, might be an effective approach for addressing cardiometabolic diseases among Southeast Asian women.

## Introduction

Cardiometabolic diseases such as cardiovascular diseases (CVD), obesity, hypertension and type 2 diabetes are a major cause of mortality, morbidity and healthcare spending worldwide [1, 2]. The prevalence of these diseases has significantly increased and has become a major problem given the significant economic burden that these diseases impose on low- and middle-income countries. Indonesia has the seventh largest number of diabetic patients (7.6 million), despite relatively low prevalence worldwide (4.8%) in 2012 [3]. In 2013, it was estimated that there were more than 132.8 million people with diabetes in the Western Pacific (more people than in any other region), and the number is expected to rise to 201.8 million by 2035 [4]. Furthermore, obesity is suggested to play a critical role in the development of chronic and non-communicable diseases (NCDs) in the Southeast (SE) Asia [5]. In Indonesia, NCDs are estimated to account for 73% of all deaths [6] of which, CVD contributed to 35% followed by cancers (12%) and diabetes (6%) [6].

Indonesia is the largest island country in the world, consisting of various ethnic groups distributed over 33 provinces [7]. Minangkabau community is the world’s largest matrilineal society which resides mostly in West Sumatra, where the prevalence of low level of high-density lipoprotein cholesterol (HDL-C), hypertension and central obesity is more than 50% [7]. It is reported that the Minangkabau ethnic group had a high risk of dyslipidemia, which is suggested to be driven mainly by the high intake of dietary fat from poor quality sources [8]. A study comparing lipid profiles among four ethnic groups reported that the Minangkabau ethnic group has the highest levels of plasma total cholesterol and low-density lipoprotein cholesterol (LDL-C) compared to other larger ethnicities including Sundanese, Javanese and Buginese [9]. Furthermore, it has been reported that the prevalence of central obesity is high among Minangkabau women [10]. Many environmental exposures contribute to the increasing prevalence of cardiometabolic diseases, but one key factor is urbanisation [11]. Countries in SE Asia have undergone rapid epidemiological and nutritional transitions over the past few decades. Furthermore, it has been reported that dietary risks, high blood pressure and tobacco smoking are the three major risk factors contributing to disease burden in Indonesia [12]. However, genetic factors also play an important role in the development of cardiometabolic diseases.

Candidate gene studies and genome-wide association studies (GWAS) have identified several single nucleotide polymorphisms (SNPs) relating to cardiometabolic diseases and traits in the Asian populations [13–16]. Most cardiometabolic traits are influenced by thousands of SNPs each having a relatively small effect on the trait when present alone. Thus, analysing the combined effects of multiple gene variants might provide a better understanding of trait variability of an individual and improve risk prediction of cardiometabolic diseases than using a single variant approach [17]. Furthermore, studies have found that the effect of genetic variants on cardiometabolic traits can be influenced by lifestyle factors [18]. It has been confirmed that using genetic risk score (GRS) approaches increases the power to detect gene-lifestyle interactions compared to the common single variant methods [19]. Therefore, our study aimed to investigate the association of a novel GRS with cardiometabolic traits and to examine whether lifestyle factors such as dietary intake and physical activity modified these associations in 110 Minangkabau women.

## Methods

### Study participants

The study included healthy women who were enrolled in the Minangkabau Indonesia Study on Nutrition and Genetics (MINANG) study, a cross-sectional pilot study conducted in the city of Padang, West Sumatra, Indonesia, between December 2017 and January 2018. This study is a part of the ongoing GeNuIne (gene-nutrient interactions) Collaboration, which aims to examine the interactions between genetic and dietary factors (nutrigenetics) on cardiometabolic disease and its related traits using population-based studies from several ethnic groups [20]. The methodology of the study has been published elsewhere [21]. In brief, 133 women were recruited from community health centres in two sub-districts in Padang City including Padang Timur and Kuranji districts to represent both urban and rural areas of Padang population, respectively. The inclusion criteria included healthy women, aged 25–60 years old and with Minangkabau ethnicity. Of the 133 enrolled women, 10 were excluded from the study according to the following exclusion criteria: being pregnant or lactating (*N* = 0) and taking dietary or vitamin supplements (*N* = 0); have a previous history of hypertension, CVD or type 2 diabetes (*N* = 6); have a body mass index (BMI) of more than 40 kg/m^2^ or being classified as morbidly obese by a practitioner (*N* = 0); being blood related to other participants in the study (*N* = 0); have any communicable disease (*N* = 4). Of the remaining 123 participants, we excluded another 5 women who did not undergo blood sampling. Thus, the final sample consisted of 118 participants, of whom seven women did not have complete genetic information about all the investigated SNPs and were excluded from the GRS analysis (*N* = 111). Additionally, one participant with no dietary information available was excluded from the GRS interaction analysis (*N* = 110).

The MINANG study was conducted according to the principles of the Declaration of Helsinki and was approved by the Ethical Review Committee of the Medical Faculty, Andalas Univesity (No.311/KEP/FK/2017). All participants gave their written informed consent before participating and had the right to withdraw from the study at will and opt-out from any of the procedures.

### Anthropometric measures

Body weight (to the nearest 100 g) and height (to the nearest mm) were measured using an electronic scale (Seca 803, Seca GmbH. Co. kg, Hamburg, Germany) and a wall-mounted stadiometer (OneMed Medicom stature meter, YF.05.05. V.A.1022, Indonesia), respectively. BMI was calculated as weight (kg)/height (m)^2^ and categorised according to the Asia-Pacific classification of BMI [22]. Waist circumference (WC) was measured in centimetre using a metal tape (Medline-OneMed Medicom, Jakarta, Indonesia) midway between the 12th rib and the superior border of the iliac crest at the end of normal expiration.

### Biochemical and clinical measures

After 12 h of fasting, blood samples (5 ml) were taken to measure the concentrations of glucose, insulin, glycated haemoglobin A1c (HbA1c), total cholesterol, triglycerides, LDL-C and HDL-C. Samples were assayed using the xMark Microplate Spectrophotometer (Bio-Rad Laboratories Inc, Hercules, California, USA). Fasting glucose, insulin and HbA1c were measured using enzyme-linked immunosorbent assay (ELISA) kits from Bioassay Technology Laboratory (Shanghai, China). Blood lipids were analysed using enzymatic colorimetric procedures, namely GPO-PAP for triglycerides and CHOD-PAP for total cholesterol, LDL and HDL. A sphygmomanometer was used to measure systolic and diastolic blood pressures (SBP and DBP). Measurements were taken twice at 5-min intervals, and the average was recorded.

### Assessment of dietary intake and physical activity

Information about dietary intake and physical activity was collected by a well-trained nutritionist in the home or in an integrated health service post. Diet was assessed using a previously validated and published semi-quantitative food frequency questionnaire (SQ-FFQ) consisting of a list of 223 food items [23]. Briefly, participants were asked to report the frequency of consumption (number of times per day, week or month) and portion size of various food items. Participants were provided with portion size images of all relevant foods to enhance reporting accuracy while completing the SQ-FFQ [24]. All collected data were double-checked for accuracy and analysed with the Indonesian Food Database and Nutrisurvey (EBISpro, Germany) to estimate total energy and macronutrient intake. Values of nutrient intake were adjusted for total energy intake using the nutrient (energy-adjusted) residual method, wherever appropriate [25].

“The Global Physical Activity Questionnaire” (GPAQ) was used to calculate an individual’s level of physical activity in 3 areas (work, transport and leisure-time) and time spent in sedentary behaviour [26]. Total time spent in moderate-to-vigorous physical activity was estimated using to the World Health Organization (WHO) STEPwise method and was expressed as metabolic equivalent minutes per day (METmins/day). Participants were defined as “active” if they did ≥ 600 METmins/week or “inactive” if they accumulated < 600 METmins/week.

### SNP selection and genotyping

Fifteen genetic variants located at 8 different genes were selected for the present study based on its consistent associations with cardiometabolic traits in candidate gene studies and GWAS in Asian populations [13–16, 27–36]. The selected genetic variants were Calpain 10 (*CAPN10*) rs3792267 and rs5030952; fat mass and obesity-associated (*FTO*)- rs9939609, rs10163409 and rs8050136; melanocortin 4 Receptor (*MC4R*)- rs17782313 and rs2229616; transcription factor 7-like 2 (*TCF7L2*)- rs12255372 and rs7903146; potassium voltage-gated channel subfamily Q member 1 (*KCNQ1*)- rs2237895 and rs2237892; cyclin-dependent kinase inhibitor 2A/2B (*CDKN2A/*2B)- rs10811661; peroxisome proliferator-activated receptor gamma (*PPARG*)- rs1801282; and adiponectin (*ADIPOQ*)-rs266729 and rs17846866.

Genomic DNA was extracted from peripheral blood leukocytes using the PureLink Genomic DNA Mini Kit (Invitrogen, Carlsbad, USA). Furthermore, a NanoDrop spectrophotometer was used to determine DNA concentration. The SNPs were genotyped using the competitive allele-specific PCR-KASP® assay at LGC Genomics (http://www.lgcgroup.com/services/genotyping).

### Statistical analysis

Statistical analysis was performed using the SPSS software (version 23). Common obesity was defined based on the Asia-Pacific classification of BMI for Asians, where non-obese individuals (BMI < 23 kg/m^2^) and obese individuals (BMI ≥ 23 kg/m^2^) were classed accordingly [37]. Central obesity was defined based on WHO classification of WC (WC > 80 cm for women) [38]. The Hardy-Weinberg equilibrium (HWE) was assessed using the *x*^2^ goodness-of-fit test, and the 15 SNPs were in HWE (*P* > 0.05). Normality of distribution of all continuous variables was tested using the Shapiro-Wilk test and those that were not normally distributed were natural log-transformed before the analysis, including glucose, insulin, HbAC1, HDL-C, LDL-C, total cholesterol, triglyceride concentrations and total dietary protein intake (%). Continuous variables are expressed as means and standard deviations (SD), and comparisons between groups were made using the independent *t* test. The descriptive statistics for categorical variables, such as physical activity level, were obtained by determining frequency distributions and compared between individuals with and without central obesity using Pearson’s chi-squared test. The association between individual SNPs and cardiometabolic traits was analysed using general linear models adjusted for age, residential area (rural or urban) and BMI when BMI is not an outcome. As the number of individuals with rare homozygous genotypes was low, a dominant model was used, where common homozygous genotypes were compared against combined rare homozygous and heterozygous genotypes.

A GRS was constructed based on 15 SNPs from 8 genes. An additive genetic model was assumed for each gene variant, assigning a score of 0, 1 and 2 to genotypes containing 0, 1 or 2 risk alleles, respectively. The GRS was then calculated for each individual by summing the number of risk alleles in the genetic variants. The count method assumed that each risk allele contributes equally and independently to the development of cardiometabolic traits. The average number of risk alleles per individual for the GRS was 5.12 (SD = 2.06), which ranged from 2 to 10. The GRS variable was then categorised into two groups based on the median of risk alleles: “low genetic risk group”—individuals with a GRS ≤ 5 risk alleles (*N* = 69) and “high genetic risk group”—individuals with GRS > 5 risk alleles (*N* = 42). The effects of GRS on cardiometabolic traits were analysed using general linear models. Furthermore, GRS-lifestyle interactions on continuous outcomes were tested using linear regression models by including the interaction terms (e.g. diet*genotype) in these models. Models were adjusted for age, residential area and additionally for BMI when it is not an outcome. Lifestyle factors that were investigated in our study included dietary intake and physical activity. Carbohydrate, protein and fat intakes were expressed as a percentage of total energy intake, and fibre intake was expressed in grammes. Furthermore, statistically significant interactions were investigated in more depth, where individuals were stratified by the tertiles of dietary intake and the levels of physical activity. A *P* value of < 0.05 was considered statistically significant. Multiple testing correction was not applied given that we had examined only one genetic instrument (i.e. GRS).

## Results

### Characteristics of the study participants according to the central obesity status

In the present study, 71 women (64.0%) were centrally obese and 39 (35.1%) were not. The characteristics of the participants are shown in Table 1. In general, centrally obese participants were older and had higher SBP (*P* = 0.006), fasting plasma glucose (*P* = 0.039), serum triglycerides (*P* < 0.001), serum total cholesterol (*P* < 0.001) and LDL-C (*P* < 0.001) concentrations compared to participants without central obesity. There were no significant differences in fasting HDL-C, serum insulin, HbA1c, DBP, dietary intake and physical activity levels and the distribution of GRS between the two groups (*P* > 0.05).

**Table 1:** Anthropometric and biochemical characteristics of the study participants

	*N*	Total (*N* = 111)	*N*	Non-centrally obese (WC ≤ 80 cm) (*N* = 39)	*N*	Centrally obese (WC > 80 cm) (*N* = 71)	*P* value*
Age (years)	111	40.49 ± 10.18	39	37.08 ± 11.68	71	42.58 ± 8.62	0.012
BMI (kg/m^2^)	111	25.13 ± 4.2	39	21.85 ± 3.71	71	26.99 ± 3.24	< 0.001
WC (cm)	110	83.85 ± 10.27	39	72.79 ± 6.03	71	89.92 ± 6.26	< 0.001
Glucose (mg/dl)	111	92.53 ± 20.67	39	87.21 ± 9.78	71	95.69 ± 24.29	0.039
Insulin (mIU/L)	111	32,428.5 ± 25,706.13	39	31,073.79 ± 28,460.35	71	33,374.28 ± 24,368.83	0.657
HbA1c (ng/ml)	111	655.59 ± 601.59	39	629.22 ± 671.07	71	666.42 ± 568.14	0.759
Triglycerides (mg/dl)	111	98.8 ± 43.47	39	78.26 ± 34.19	71	109.72 ± 44.38	< 0.001
Cholesterol (mg/dl)	111	209.31 ± 44.02	39	188.26 ± 30.04	71	221.77 ± 45.74	< 0.001
HDL-C (mg/dl)	111	59.12 ± 10.29	39	60.9 ± 10.45	71	58.14 ± 10.22	0.182
LDL-C (mg/dl)	111	128.12 ± 39.85	39	111.49 ± 25.55	71	138.2 ± 42.65	< 0.001
SBP (mmHg)	111	113.37 ± 9.07	39	110.14 ± 8.83	71	115.05 ± 8.81	0.006
DBP (mmHg)	111	77.44 ± 6.39	39	76.26 ± 8.35	71	78.06 ± 5.01	0.223
Total energy (kcal/day)	110	1776.24 ± 611.43	39	1789.55 ± 604.31	70	1755.6 ± 613.59	0.781
Carbohydrate intake (%)	110	53.97 ± 9.44	39	52.67 ± 7.86	70	54.91 ± 10.1	0.235
Protein intake (%)	110	16.93 ± 3.32	39	17.13 ± 2.93	70	16.76 ± 3.54	0.579
Fat intake (%)	110	28.95 ± 7.99	39	30.05 ± 6.87	70	28.16 ± 8.45	0.235
Dietary fibre (g)	110	8.78 ± 4.29	39	9.11 ± 4.52	70	8.56 ± 4.19	0.521
SFA (g)	110	20.84 ± 11.22	39	21.77 ± 10.81	70	20.07 ± 11.35	0.447
MUFA (g)	110	8.18 ± 4.6	39	9.00 ± 5.08	70	7.62 ± 4.18	0.129
PUFA (g)	110	6.32 ± 3.5	39	6.67 ± 3.06	70	6.14 ± 3.76	0.541
MET (min/week)	111	1311.89 ± 1877.78	39	1114.87 ± 1625.95	71	1428.45 ± 2016.27	0.407
GRS	110	5.09 ± 2.07	39	4.77 ± 2.01	71	5.31 ± 2.03	0.189
Physical activity levels	44	Sedentary (39.64%)	18	Sedentary (46.15%)	26	Sedentary (36.62%)	0.616
	55	Moderate (49.55%)	17	Moderate (43.59%)	37	Moderate (52.11%)	
	12	Vigorous (10.81%)	4	Vigorous (10.26%)	8	Vigorous (11.27%)	

Data presented as means ± SD for continuous variables and as percentages for categorical variables

*BMI* body mass index, *WC* waist circumference, *HbA1C* glycated haemoglobin A1c, *HDL-C* high-density lipoprotein cholesterol, *LDL-C* low-density lipoprotein cholesterol, *SBP* systolic blood pressure, *DBP* diastolic blood pressure, *SFA* saturated fatty acids, *MUFA* monounsaturated fatty acids, *PUFA* polyunsaturated fatty acids, *MET* metabolic equivalent of task, *GRS* genetic risk score

*P values for the differences in the means and proportions between non-centrally obese and centrally obese individuals were calculated using the independent *t* test and the Chi-squared test, respectively

### Associations between GRS and cardiometabolic traits

To explore the combined effect of the 15 SNPs on various cardiometabolic traits, a GRS was calculated. There was a significant association (*P* = 0.018) between the GRS and BMI where individuals carrying 6 or more risk alleles of the SNPs had higher BMI compared with those carrying 5 or less risk alleles (Table 2).

**Table 2:** Associations between GRS and cardiometabolic traits

	GRS ≤ 5 (*N* = 69)		GRS > 5 (*N* = 42)		*P* value*
	*N*	Mean ± SE	*N*	Mean ± SE	
BMI (kg/m^2^)	69	24.52 ± 0.52	42	26.14 ± 0.6	0.018
WC (cm)	68	84.28 ± 1.22	42	83.16 ± 1.66	0.334
Log glucose (mg/dl)	69	93.65 ± 2.98	42	90.69 ± 1.72	0.327
Log insulin (mIU/L)	69	32,365.29 ± 3199.95	42	32,532.33 ± 3782.96	0.196
Log HbA1C (ng/ml)	69	650.58 ± 71.1	42	663.81 ± 96.65	0.527
Log triglycerides (mg/dl)	69	101.07 ± 5.27	42	95.07 ± 6.67	0.142
Log cholesterol (mg/dl)	69	212.88 ± 5.59	42	203.43 ± 6.11	0.228
Log HDL-C (mg/dl)	69	58.55 ± 1.26	42	60.05 ± 1.56	0.404
Log LDL-C (mg/dl)	69	131.84 ± 4.97	42	122 ± 5.73	0.197
Log SBP (mmHg)	69	113.12 ± 1.08	42	113.77 ± 1.43	0.679
Log DBP (mmHg)	69	77.59 ± 0.86	42	77.2 ± 0.76	0.535

*BMI* body mass index, *WC* waist circumference, *HbA1C* glycated haemoglobin A1c, *HDL-C* high-density lipoprotein cholesterol, *LDL-C* low-density lipoprotein cholesterol, *SBP* systolic blood pressure, *DBP* diastolic blood pressure

*P values obtained from linear regression analysis adjusted for age, residential area and additionally for BMI when BMI is not an outcome. The analysis was performed on log-transformed variables

### Interactions between GRS and dietary intake on cardiometabolic traits

**Fig. 1:**
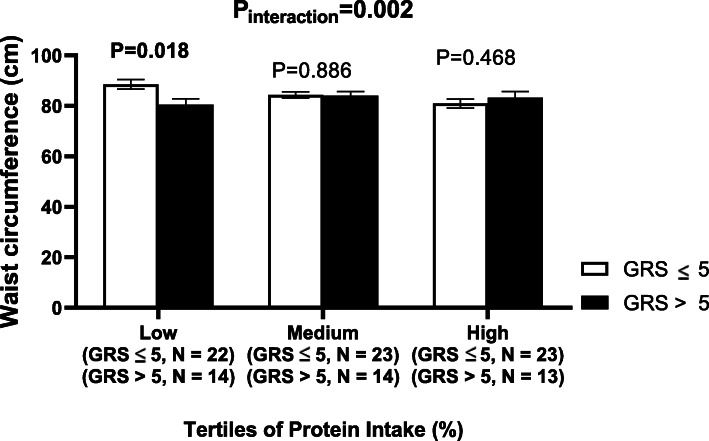
Interaction between genetic risk score (GRS) and log protein intake (%) on waist circumference (WC). White bars indicate “low genetic risk group”: individuals with a GRS ≤ 5 risk alleles; black bars indicate “high genetic risk group”: individuals with GRS > 5 risk alleles. Carriers of 6 or more risk alleles had lower WC compared to carriers of 5 or less risk alleles, among individual with lower protein intake (13.51 ± 1.18%)

**Fig. 2:**
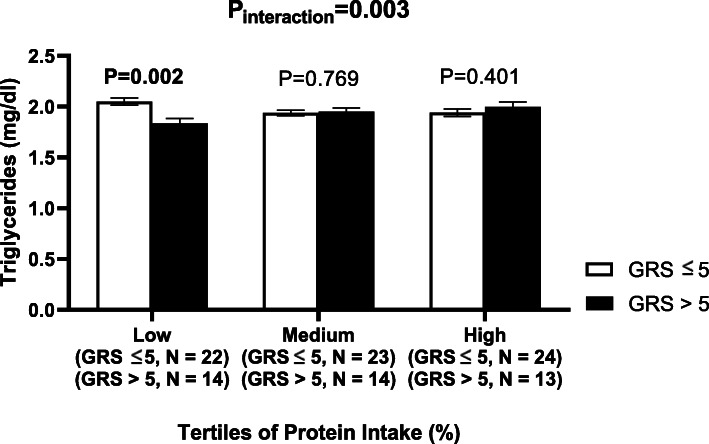
Interaction between genetic risk score (GRS) and log protein intake (%) on log triglyceride levels. White bars indicate “low genetic risk group”: individuals with a GRS ≤ 5 risk alleles; black bars indicate “high genetic risk group”: individuals with GRS > 5 risk alleles. Carriers of 6 or more risk alleles had lower triglyceride level compared to carriers of 5 or less risk alleles, among individual with lower protein intake (13.51 ± 1.18%)

There were significant interactions between the GRS and protein intake (%) on WC and triglyceride concentrations (*P*_interaction_ = 0.002 and 0.003, respectively) (Table 3). With low protein intake (13.51 ± 1.18%), carriers of 6 or more risk alleles of SNPs had lower WC and triglyceride concentration compared to carriers of 5 or less risk alleles (*P* = 0.0118 and 0.002, respectively) (Figs. 1 and 2). A significant interaction between protein intake and GRS was also detected on cholesterol levels (*P*_interaction_ = 0.021). Moreover, there were no other interactions between nutrient intake and GRS on cardio-metabolic traits.

**Table 3:** Interactions between GRS and lifestyle factors on cardio-metabolic traits

	Carbohydrate (%)	Protein (%)	Fat (%)	Fibre (g)	Physical activity
BMI (kg/m^2^)	0.961	0.282	0.721	0.876	0.362
WC (cm)	0.224	**0.002**	0.577	0.614	0.297
Log glucose (mg/dl)	0.882	0.751	0.732	0.833	0.106
Log insulin (mIU/L)	0.336	0.341	0.48	0.216	0.909
Log HbA1C (ng/ml)	0.766	0.638	0.935	0.162	0.626
Log triglycerides (mg/dl)	0.066	**0.003**	0.355	0.262	0.479
Log cholesterol (mg/dl)	0.081	0.021	0.261	0.583	0.308
Log HDL-C (mg/dl)	0.978	0.905	0.984	0.323	0.540
Log LDL-C (mg/dl)	0.266	0.337	0.431	0.896	0.721
Log SBP (mmHg)	0.156	0.291	0.208	0.872	0.644
Log DBP (mmHg)	0.966	0.815	0.732	0.292	0.743

Data are *P* values obtained from linear regression analysis adjusted for age, residential area and BMI when BMI is not an outcome. The analysis was performed on log-transformed variables

*BMI* body mass index, *WC* waist circumference, *HbA1C* glycated haemoglobin A1c, *HDL-C* high-density lipoprotein cholesterol, *LDL-C* low-density lipoprotein cholesterol, *SBP* systolic blood pressure, *DBP* diastolic blood pressure

### Associations between individual SNPs and cardiometabolic traits

As shown in supplementary Table 1, Additional File 1, we found that the risk alleles of the three *FTO* SNPs rs9939609, rs8050136 and rs10163409 were associated with higher BMI (*P* = 0.006, 0.007 and 0.047, respectively). Furthermore, SNPs rs12255372 (*TCF7L2*), rs2237892 (*KCNQ1*) and rs5030952 (*CAPN10*) were associated with increased fasting serum LDL-C concentrations (*P* = 0.032, 0.039 and 0.04, respectively). A significant association was also found between the risk allele of the SNP rs17782313 (*MC4R*) and higher insulin level (*P* = 0.036). No significant association was observed between the remaining SNPs and cardiometabolic traits in this population (*P* > 0.05).

## Discussion

The present study aimed to investigate the effects of genetic predisposition and lifestyle factors on cardiometabolic traits in Minangkabau women. In agreement with other studies [39], we have shown that the GRS based on 8 susceptible genes for cardiometabolic diseases is a significant risk factor for higher BMI in our study sample and might be a useful tool in characterising Minangkabau women at high risk for obesity. We found that women carrying 6 or more alleles had significantly higher BMI compared to those carrying 5 or less risk alleles. Furthermore, we found a significant interaction between the GRS and dietary protein intake (%) on WC and triglyceride levels, where, among those who consumed a low protein diet (mean intake ± SD 13.51 ± 1.18%), individuals, despite carrying more than 6 risk alleles, had significantly lower WC and triglyceride levels. Given that Minangkabau women have a high risk of dyslipidemia [9] and the prevalence of common and central obesity is high among this ethnic group [10], it is important to develop effective strategies targeting these conditions to improve public health.

It has been suggested that centrally obese participants defined as normal weight based on BMI had the worst long-term survival even when compared with their overweight and obese counterparts [40]. In addition, recent data from 42,702 European participants reported that central obesity is associated with higher mortality risk even in normal-weight individuals [41]. This is of concern for Asian populations, where increased levels of visceral adiposity are observed in those with normal BMIs [42–44]. Furthermore, the combination of increased WC along with elevated triglyceride levels has been previously defined as the ‘hypertriacylglycerolaemic waist’ phenotype [45]. Studies have shown that individuals with this phenotype have an increased risk of higher visceral adiposity, CVD, insulin resistance and other related outcomes [45]. Therefore, targeting this phenotype will have significant public health implications in terms of reducing NCD mortality in Asian populations.

In the present study, the average protein intake was 77 ± 37 g/day, which exceeded the recommended dietary protein daily allowance of 57–59 g/day for Indonesian women [46, 47]. Observational studies have shown that higher protein intake was significantly associated with increases in body weight, BMI and fat mass [48–50]. These results are in contrast to the finding from intervention studies, which have shown that high protein intake enhances weight loss and provides a better long-term maintenance of reduced intra-abdominal fat stores [37, 51]. These inconsistencies might be attributed to the sample size, genetic heterogeneity and gene-lifestyle interactions. Cross-sectional studies have demonstrated the association of several SNPs with obesity-related traits [52–55], and interaction of these SNPs with dietary intake of protein on weight change [56–58]. It has been shown that high protein diets can modulate the genetic effect of *FTO* variants on body weight, BMI and WC [59–61]. According to a 2-year weight loss intervention programme, carriers of the risk allele ‘A’ of the *FTO* SNP rs1558902 had a greater reduction in weight and regional fat compared to non-carriers when high protein diets were consumed, whereas an opposite genetic effect was found on changes in fat distribution in response to a low-protein intake [60]. However, studies investigating the joint effect of genetic variants have reported conflicting results [62–64], indicating that the influence of genetic predisposition on changes in body weight and WC does not seem to be modulated by protein intake. In contrast, the present study provides evidence for GRS-protein intake interactions on WC and triglyceride concentrations, and these interactions were independent of potential confounding effects. We found that participants with 6 or more risk alleles who consumed a low protein diet (mean intake ± SD 13.51 ± 1.18%) had significantly lower WC and triglyceride concentrations compared to those with 5 or less risk alleles. This difference in the findings across the studies might be due to differences in the sample size, methods used to construct GRSs (weighted vs. unweighted) and the number of SNPs included in the GRSs.

The observed interaction between GRS and dietary protein on WC and triglyceride concentrations might be driven by the source of protein consumed, which has not been analysed in our study. Different protein sources have different effects on body weight and fat mass, and the mechanisms behind this are still very speculative and need more investigation. The higher intake of protein from animal sources (protein from red and processed meat and poultry) was found to be associated with an increase in body weight in both genders, with a stronger association in women [49]. Diet rich in animal protein might reflect the western pattern diet characterised by high red meat consumption, which has shown to be associated with weight gain [65]. In contrast, a study has shown that protein from meat is associated with lower weight gain because it produces a higher 24-h energy expenditure compared to soy protein [66]. This hypothesis is, however, based on a mechanistic study, and it is still unknown whether this applies in the long run to individuals of the free-living populations. Furthermore, it has been suggested that consuming protein from dairy sources may prevent weight gain and promote abdominal fat loss [67]. Here, the suggested mechanism primarily relates to the high content of calcium, which may function synergistically in combination with bioactive compounds, such as angiotensin-converting enzyme inhibitors and the rich concentration of branched-chain amino acids [67]. While the above-mentioned studies failed to explore the genetic aspects, our study did not investigate the type of protein that was consumed by the participants; hence, future studies examining the effect of both factors are required.

In agreement with some studies [62, 63], no interactions were detected between GRS and dietary intake of protein, fat and carbohydrate on BMI in the present study. However, a study in the European population (*N* = 48,170 adults) has shown that the joint effect of 93 obesity-related SNPs on BMI might be modulated by the intake of total energy, fat and saturated fat [64]. Furthermore, studies have shown that an obesogenic diet and physical inactivity with relatively high intake of sugar-sweetened beverages and prolonged television watching might exaggerate the effect of genetic factors on adiposity [18, 68]. Even though several studies have demonstrated that physical activity could attenuate the combined genetic influence of multiple SNPs on BMI and obesity risk [18, 69, 70], no such interactions were detected in the present study.

The strengths of our study include the use of a well-defined population, a validated SQ-FFQ [23] and a genetic risk score generated from the 15 genetic variants associated with cardiometabolic traits. Also, the main exposures investigated in our study were collected by well-trained staff and using validated and standardised operating procedures. However, there are limitations that need to be acknowledged. Although our analysis was adjusted for several factors, the potential for confounding by unmeasured or unknown factors exist. Even though our study has a small sample size, we were still able to find significant associations and interactions suggesting that our study is well powered. Even though food intake was assessed using validated methods, recall bias and measurement errors in these self-reported FFQs cannot be fully eliminated, which could alter the true underlying interactions between dietary and genetic factors on cardiometabolic traits [71, 72]. Finally, our study was restricted to Minangkabau women, and it is unknown whether our findings could be generalised to men or other demographic or ethnic groups.

## Conclusion

In the present study, we have shown a significant effect of the GRS on WC and triglyceride levels through the influence of a low protein intake, where individuals with a high genetic susceptibility can overcome the risk of higher WC and triglyceride levels by consuming a low protein diet. These findings are potentially relevant for public health; however, future trials in both genders with larger sample size and objective measures of protein intake, such as urinary nitrogen, are needed to confirm these findings.

## Supplementary information

**Additional file 1:.** Supplementary Table 1 Associations between individual SNPs and cardiometabolic traits.

## Data Availability

The datasets used and/or analysed during the current study are available from the corresponding author on reasonable request.
