# Supplementary material for: Interaction between the genetic risk score and dietary protein intake on cardiometabolic traits in Southeast Asian
Source: Genes Nutr. 2020 Oct 12;15:19. doi: 10.1186/s12263-020-00678-w (PMC7552350; doi:10.1186/s12263-020-00678-w)
Supplement: Supplementary file 1 — Additional file 1:. Supplementary Table 1 Associations between individual SNPs and cardiometabolic traits. [file 12263_2020_678_MOESM1_ESM.docx]

**Supplementary Table 1** Associations between individual SNPs and cardiometabolic traits.

|  |  | BMI (kgm2) | WC (cm) | Log Glucose (mg/dl) | Log Insulin (mIU/L) | Log HbA1C (ng/ml) | Log triglycerides (mg/dl) | Log Cholesterol (mg/dl) | Log HDL-C (mg/dl) | Log LDL-C (mg/dl) | Log SBP (mmHg) | Log DBP (mmHg) |
| --- | --- | --- | --- | --- | --- | --- | --- | --- | --- | --- | --- | --- |
| *MC4R* | rs17782313 | 0.967 | 0.552 | 0.969 | 0.036 | 0.135 | 0.238 | 0.234 | 0.565 | 0.595 | 0.447 | 0.928 |
| *MC4R* | rs2229616 | 0.81 | 0.968 | 0.691 | 0.301 | 0.35 | 0.522 | 0.846 | 0.565 | 0.783 | 0.52 | 0.945 |
| *FTO* | rs9939609 | 0.006 | 0.776 | 0.327 | 0.553 | 0.605 | 0.751 | 0.961 | 0.685 | 0.743 | 0.982 | 0.517 |
| *FTO* | rs8050136 | 0.007 | 0.754 | 0.301 | 0.558 | 0.632 | 0.805 | 0.867 | 0.639 | 0.813 | 0.882 | 0.639 |
| *FTO* | rs10163409 | 0.047 | 0.833 | 0.18 | 0.985 | 0.731 | 0.182 | 0.411 | 0.969 | 0.431 | 0.749 | 0.629 |
| *TCF7L2* | rs7903146 | 0.645 | 0.115 | 0.762 | 0.439 | 0.19 | 0.639 | 0.58 | 0.666 | 0.097 | 0.762 | 0.423 |
| *TCF7L2* | rs12255372 | 0.722 | 0.152 | 0.801 | 0.316 | 0.164 | 0.543 | 0.276 | 0.692 | 0.032 | 0.538 | 0.915 |
| *ADIPOQ* | rs266729 | 0.837 | 0.59 | 0.275 | 0.581 | 0.957 | 0.258 | 0.682 | 0.774 | 0.654 | 0.693 | 0.274 |
| *ADIPOQ* | rs17846866 | 0.221 | 0.555 | 0.21 | 0.129 | 0.179 | 0.797 | 0.597 | 0.774 | 0.845 | 0.882 | 0.389 |
| *KCNQ1* | rs2237895 | 0.263 | 0.606 | 0.847 | 0.199 | 0.33 | 0.782 | 0.803 | 0.801 | 0.746 | 0.832 | 0.597 |
| *KCNQ1* | rs2237892 | 0.501 | 0.215 | 0.937 | 0.502 | 0.775 | 0.868 | 0.546 | 0.16 | 0.039 | 0.866 | 0.968 |
| *CDKN2A/B* | rs10811661 | 0.392 | 0.51 | 0.887 | 0.617 | 0.253 | 0.76 | 0.815 | 0.83 | 0.254 | 0.497 | 0.224 |
| *PPARG* | rs1801282 | 0.128 | 0.207 | 0.988 | 0.743 | 0.42 | 0.4 | 0.411 | 0.921 | 0.101 | 0.782 | 0.456 |
| *CAPN10* | rs3792267 | 0.977 | 0.433 | 0.664 | 0.427 | 0.889 | 0.061 | 0.374 | 0.458 | 0.964 | 0.341 | 0.47 |
| *CAPN10* | rs5030952 | 0.88 | 0.852 | 0.949 | 0.566 | 0.849 | 0.828 | 0.425 | 0.373 | 0.04 | 0.773 | 0.912 |

Data are p values obtained from linear regression analysis adjusted for age, residential area and BMI when BMI is not an outcome. Abbreviations: *FTO* Fat mass and obesity associated gene; *MC4R* Melanocortin 4 Receptor ; *TCF7L2* Transcription factor 7-like 2; *ADIPOQ* Adiponectin*; KCNQ1* Potassium voltage-gated channel subfamily Q member 1; *CDKN2A/2B* Cyclin dependent kinase inhibitor 2A/2B; *PPARG* Peroxisome proliferator-activated receptor gamma and *CAPN10* Calpain 10; BMI Body mass index; WC Waist circumference; HbA1C glycated haemoglobin A1c; HDL-C High-density lipoprotein cholesterol; LDL-C Low-density lipoprotein cholesterol; SBP Systolic blood pressure; DBP diastolic blood pressure
